# New Insights in Psychiatric Disorder Psychopharmacology

**DOI:** 10.3390/brainsci15030319

**Published:** 2025-03-19

**Authors:** Mohammadreza Shalbafan, Laura Orsolini

**Affiliations:** 1 Mental Health Research Center, Psychosocial Health Research Institute (PHRI), Department of Psychiatry, School of Medicine, Iran University of Medical Sciences, Tehran 14496-14535, Iran; shalbafan.mr@iums.ac.ir; 2 Brain and Cognition Clinic, Institute for Cognitive Sciences Studies, Tehran 14496-14535, Iran; 3 Unit of Clinical Psychiatry, Department of Neurosciences/DIMSC, Polytechnic University of Marche, 60126 Ancona, Italy

Psychopharmacological interventions are the treatment cornerstone of most psychiatric disorders such as depression, schizophrenia, obsessive–compulsive disorder, and bipolar disorders, with a huge number of studies including clinical trials and review articles deepening knowledge in the field in recent decades [1,2,3]. In recent years, novel proposed mechanisms for medication actions for psychiatric disorders, as well as novel medications targeting new paths such as glutamatergic and inflammatory systems, have been investigated substantially [4,5,6,7,8]. In light of this fact, the current Special Issue calls for new insight in psychopharmacology for the treatment of psychiatric disorders to enrich the literature in this topic, publishing nine papers, which are briefly described in this editorial.

Four papers investigated different aspects of psychopharmacological treatments of schizophrenia. Dao et al. conducted a Systematic Review on the effect of Aspirin on cardiovascular events in patients with schizophrenia and concluded that although the evidence is growing in favor of the useful effect of this medication in this vulnerable group, the current literature is not conclusive and further rigor studies are needed (contribution 1). Another Review Article by Messina and his colleagues investigated the effect of anti-inflammatory and neuro-protective medications on schizophrenia. The authors comprehensively reviewed trials on different anti-inflammatory agents in this group of patients and concluded that they are useful as an adjuvant therapy. In addition, they suggested inflammatory screening for patients with schizophrenia, particularly in the first episode (contribution 2). Furthermore, Tsapakis et al. conducted a Selective Review on novel treatments of schizophrenia on different neurobiological targets such as dopamine, serotonin, glutamate, acetylcholine, and trace amines and concluded that further studies should target different aspects of this complex disorder, more specifically the debilitating negative and cognitive symptoms (contribution 3). Moreover, de Filippis and his colleagues conducted a prospective naturalistic study on the effectiveness of second-generation antipsychotics-long-acting injections (SGA-LAIs) on clinical, cognitive, and social domains of schizophrenia. They suggested that switching from oral SGA to SGA-LAIs (in their study, aripiprazole monohydrate (Ari-LAI) and paliperidone palmitate 1 and 3 months (PP1M, PP3M)) represents a valid and effective treatment strategy, with significant improvements in psychopathological, cognitive, social, and clinical variables for patients suffering from schizophrenia (contribution 4).

On the other hand, three papers dealt with pharmacological treatments for depression. Dogaru et al. reviewed the perspective on the effect of pharmacological and non-pharmacological treatments via anti-inflammatory mechanisms among patients with unipolar depression. They concluded that both conventional and non-conventional anti-inflammatory drugs showed promising results according to the specific group of patients. The pre-existing pro-inflammatory status was, in most cases, a predictor for clinical efficacy and, in some cases, a correlation between clinical improvement and changes in various biomarkers was found. Some of the non-pharmacological interventions (physical exercise and electroconvulsive therapy) have also shown beneficial effects for depressive patients with elevated inflammatory markers (contribution 5). In addition, Alvarez-Mon and colleagues surveyed the perspective of psychiatrists in Spain on Duloxetine and concluded, based on 163 responses, that there is agreement regarding the use of duloxetine, not only due to its tolerability and effectiveness but also due to the wide variety of situations in which it can be used (e.g., somatic symptoms in fibromyalgia, diabetes) as it also relieves neuropathic pain (contribution 6). Lalegani and her colleagues also conducted an 8-week open-labeled clinical trial to investigate the efficacy and safety of drug holiday among female patients with selective-serotonin reuptake inhibitors (SSRIs)-induced sexual side effects. Their trial showed the drug holidays’ group showed significant improvements in arousal, desire, orgasm, satisfaction, lubrication, and overall sexual health. Drug holidays did not introduce immediate safety concerns or significant adverse effects (contribution 7). Furthermore, Nohesara et al. published a Review article entitled ‘Substance-Induced Psychiatric Disorders, Epigenetic and Microbiome Alterations, and Potential for Therapeutic Interventions’ and comprehensively reviewed the topic, suggesting further investigations on the connection between different aspects (contribution 8). Last but not least, Ragnhildstveit and colleagues reviewed the evidence for the effectiveness of Ketamine in eating disorders and concluded that overall, results are encouraging, and point to its therapeutic value; however, the results are limited to case series and reports on anorexia nervosa; therefore, further empirical research is needed (contribution 9). This Special Issue is now freely available for all basic and clinical investigators hoping for a better understanding of psychopharmacology as one of the fastest growing aspects of psychiatric treatments.
